# EU wine policy in the framework of the CAP: post-2020 challenges

**DOI:** 10.1186/s40100-020-00159-z

**Published:** 2020-06-16

**Authors:** Eugenio Pomarici, Roberta Sardone

**Affiliations:** 1 Dipartimento Territorio e Sistemi Agro-forestali (TeSAF), Università di Padova, Padua, Italy; 2 CREA – Centro di Ricerca Politiche e Bio-economia, Rome, Italy

**Keywords:** CAP reform, Wine sustainability, CAP Strategic Plan, EU wine sector performance

## Abstract

The EU Common Agricultural Policy (CAP), and with it the EU wine policy, is experiencing a reform process, started in 2018, in order to address ambitious environmental and social objectives, in conjunction with the goal of a competitive agricultural sector.

Given the role of the EU in wine supply, the aim of this paper is to present the design, the rationale and the potential effect of the proposed reform with specific reference to wine sector. To better understand the forthcoming process, it is firstly presented how CAP and its wine policy evolved in terms of objectives and tools over time.

The EU wine policy is a paradigmatic example of a combination between the horizontal measures, valid for all agricultural sectors, and vertical measures, peculiarly encompassing the whole wine supply chain. The reform proposal confirms, with some interesting modifications, the set of tools already operating in the sector; however, it calls for a planning of the implementation of the available tools for all products in a unitary frame represented by a national CAP Strategic Plan, applying a lean administrative procedure.

In the hypothesis that the COVID-19 outbreak will not cause a radical change in the global agri-food system, the proposed planning process should stimulate shared strategies. These are intended to effectively coordinate, according to the principle of complementarity, the implementation of available policy tools, in order to obtain a better use of resources and a more balanced achievement of all policy objectives.

## Introduction

In 2018, the reform process of the Common Agricultural Policy (CAP) started with the aim to define a new governance model for the post-2020 European agriculture. The CAP reform process will take place simultaneously with the reshaping of rules for the management of the Multiannual Financial Framework (MFF), and the UK exit from the European Union (EU). The MFF revision appears to be a significant one, both from the perspective of resources to finance the future common budgets and the perspective of spending-policy priorities supported by the EU. All official documents produced by European institutions highlight the urgency to launch a new season of common policies more capable of addressing both the new challenges regarding environmental protection and climate mitigation and the deep social, economic and international tensions that Europe is experiencing (European Commission 2016a, 2017a, 2018a, 2019a). With regard to agriculture, the European Commission proposal regarding the MFF of May 2018 has planned a general reduction in the amount of resources for the CAP compared with the previous financial period, as a result of different levels of cuts suffered by the two pillars of the CAP, with rural development making the largest contribution to saving resources (Agrafacts 2018; Matthews 2018; EPRS (European Parliamentary Research Service) 2018). Despite this reduction, the CAP confirms its significant role in the European budget, with a share close to 30% of the total.

The CAP reform proposal was officially presented in June 2018, with the launch of three new regulations that shape its structure after 2020 (European Commission 2018b, 2018c, 2018d). The new CAP should be focused on a general confirmation of the objectives defined in 2013, which are better stated in nine clearer and well-defined objectives.

The main novelty of the new CAP is the greater relevance assigned to the principle of subsidiarity^1^, which implies a greater role for Member States (MS) in CAP planning. This implies a new governance and a completely new management approach in policy implementation: the introduction of the CAP Strategic Plan (Erjavec 2018). This new programming document is based on a so-called “tailor-made approach”; in fact, the CAP Strategic Plan must contain an extensive analysis of specific national needs and indications regarding how each country intends to meet the overall CAP objectives. The aim is to use a single strategic framework to combine the different traditional tools of agricultural policy: direct payments to farmers, support for investment and innovation within sectoral or horizontal measures, incentives for the adoption of specific production practices, regulations, sectoral and rural development interventions, etc.

The new regulations should have been approved at the beginning of 2020, but given the deadlock in the approval of MFF, which is currently worsened by the spread of the COVID-19, the current CAP will be extended at least until 2021. However, considering that in recent months the proposal has passed through extensive consultations with stakeholders and delegations representing the MSs governments, it is also reasonable that the European institutions will approve a reform that is largely consistent with the proposals analysed here (European Parliament 2018). Moreover, the proposed new regulations should be fully consistent with the strategy “Farm to Fork”, announced for the spring 2020, regarding the implementation in the agricultural sector of the European Green Deal, the EU programme of investments for the environmental protection and the climate change mitigation.

The wine sector, which represents an important segment of EU agriculture, is also obviously involved in the CAP revision process. In relation to that, it must be underlined that a vertical policy addressed to wine (a wine policy *strictu sensu*) has been part of the CAP since the very beginning, being characterised by its own physiognomy and the identification of specific sectoral objectives, partially different from those stated for other sectoral interventions. Such peculiarity has been preserved and deepened over time through many different reform processes principally driven by a change in wine policy focus that in the last decades shifted from strict control of production in quantitative terms towards more attention to the quality and improvement of the competitiveness of European wine production in the global market (Pomarici and Sardone 2001 and Pomarici and Sardone 2009; Meloni and Swinnen 2013; Corsinovi and Gaeta 2017).

In the past, the EU sectoral wine policy has received numerous criticisms, particularly with regard to its complex structure and relatively high share of expenditure for ineffective, inefficient and contradictory interventions (such as distillation). At the beginning of this century, two reports, delivered to the European Commission and Parliament, pointed out some weaknesses and inefficiencies in the EU wine policy (INNOVA 2004; European Parliament 2006); these reports contributed to the most significant sectoral reform, approved in 2008. The recommendations highlighted by these reports were focused on reshaping the common wine policy in order to make the EU wine sector more responsive to the international market in terms of both quantity and quality of production.

Today, the EU wine policy appears profoundly different from the one that managed the sector until the early years of this century, but, despite the major changes implemented in 2008 and the further adjustments introduced by the CAP revision in 2013, the current EU wine policy is still subject of severe judgments by many academics, particularly with regard to its strict regulation and complexity. Meloni and Swinnen (2013) stressed that EU legislation is rooted in the wine regulations developed in France, Italy and Germany starting in the final years of the nineteenth century and that it has progressively affected the most important areas of global wine production. In particular, the authors wrote “As a consequence, what were initially mainly French and, to a lesser extent, Italian national regulations now apply to approximately 60% of the world’s wine production. This demonstrates how inefficient institutions and regulations can grow because of a combination of economic, political and institutional integration and the associated political pressure and influence” (p. 272). Gaeta and Corsinovi (2014) in their book about EU wine policy defined this policy as a tentacled monster and raised the question “whether the existence of the legislative labyrinth that the wine sector is lost in is justified or whether it is benefitting someone” (Preface). More forgiving is the last evaluation of wine sectoral policy made available by the European Commission in April 2019 (Agrosynergie GEIE 2018).

Nevertheless, the EU’s complex regulation of wine production cannot be considered an isolated case, even if it is probably the most structured on the world panorama. In fact, in non-European countries, the growth of the wine industry has also developed under appropriate legislation, driven by objectives related to fiscal issues, product integrity and consumer protection (Georgopoulos 2014). Moreover, the question of the need for harmonisation of different legislation is not new, given that it emerged just after the first world war, thus motivating the creation of the Office International de la Vigne et du Vin, in 1924^2^, and more recently the establishment of the World Wine Trade Group, in 1998^3^.

Actually, the proposed CAP revision for the period 2021–2027 does not substantially modify the current peculiar structure of wine policy, which is also going to suffer a relatively lower cut in its financial assignment, in comparison with the expected redistribution of resources for the overall CAP in the context of MFF revision (European Commission COM (2018) 392 final, Annex V). Anyway, the planned revision concerning some specific wine rules combined with an updated policy framework could change, or possibly improve, the effectiveness of EU support to the wine sector. Then, given the role of EU wine supply in the world scene, it will be of interest, from an academic, political and professional point of view, to understand how the institutional setting of the sector—the EU wine policy—will evolve and what the consequences for the sector’s performance could be. At the moment of writing, despite some relevant papers and books about wine policy, most of them cited in this paper, there is a lack of literature that analyses the position of EU wine policy within the CAP and how this stems from the various parts of CAP. This could make it difficult, for most observers, to have a full grasp of the functioning of the policy framework in which EU wine producers operate and the meaning of the proposed changes for the post-2020 period.

In order to offer comprehensive information that will enable a better understanding of the CAP revision process that will occur over the next months and to identify the potential consequences for the EU wine sector, the objective of this paper is twofold.

First, to synthetically present how CAP and, within CAP, the wine policy evolved both in terms of objectives and tools and to present the complex set of tools that currently define—in a broad sense—the EU’s wine policy. Then, to present the design of the reform and, with specific reference to wine, its rationale and potential effect in light of the current performance of the EU wine sector; the latter in the hypothesis that the COVID-19 outbreak will not cause a radical change of the global agri-food system and an extraordinary immediate support by EU and MSs will preserve the present structure of the EU wine industry

The rest of this paper is organised as follows: the “Evolution of the CAP and wine policy” section presents how CAP objectives and structure changed over about 70 years and how, within this framework, the wine policy consequently evolved; the “The current support to the wine sector within the CAP” section presents in detail the current wine policy architecture, highlighting the relations between objectives and tools; the “The performance of EU wine policy: an analytical evaluation” section, using a wide set of indicators, explores how the EU wine sector, considering different aspects, fulfils policy objectives; the “EU wine policy after 2020” section presents the reform project and the implications for the wine sector and explores the potential impact on the wine sector of the proposed changes; finally, the “Conclusions” section proposes some closing remarks.

## Evolution of the CAP and wine policy

The European Common Agricultural Policy, established in 1962, represents the cornerstone of the EU. It was the first common policy implemented after the signing of the Treaty of Rome (in 1957) and benefitted, up to the Multiannual Financial Framework 2000–2006, from receiving the largest part of the EU’s budget. CAP’s objectives were defined under article 39 of the Treaty of Rome, focusing on the main issues related to economic and social concerns of the agricultural sector, and the safeguarding of both producers and consumers. The objectives are listed here in their original version:

- To increase agricultural productivity by promoting technical progress and by ensuring the rational development of agricultural production and the optimum utilisation of the factors of production, in particular labour;
- To ensure a fair standard of living for the agricultural community, in particular by increasing the individual earnings of persons engaged in agriculture;
- To stabilise markets;
- To assure the availability of supplies;
- To ensure that supplies reach consumers at reasonable prices.

Nevertheless, without a formal rewriting of the Treaty, during the following decades, following the deep structural changes occurred in European agriculture^4^, several processes of reform have reshaped European interventions in agriculture and the focus of the CAP has been progressively expanded. Alongside the traditional objectives, many additional goals have gained relevance, which nowadays represent important guidelines for common intervention in the agricultural sector. These new objectives address different issues, including environmental protection, promotion of sustainable development, animal welfare, food quality and safety, consumer protection, safeguarding of employment, public health, and economic, social and territorial cohesion (Massot 2018). In more recent years, with the 2013 CAP reform for the programming period 2014–2020, the aforementioned complexity of objectives has been formally recognised with the identification of three main goals defined as guidelines for European action in agriculture: viable food production, sustainable management of natural resources and climate action, and lastly, balanced territorial development (European Commission COM(2010) 672 final; European Parliament 2015). In particular, viable production is identified as the capacity to contribute to farm income and to limit variability in that income, to improve the competitiveness of the agricultural sector and enhance its value share in the food chain (European Parliament 2017). Sustainable management of natural resources and climate action is aimed at the widespread introduction of sustainable production practices, provision of environmental public goods, promotion of green growth through innovation, and climate change mitigation. Lastly, the objective of balanced territorial development calls for improvement of the economy and social conditions of rural areas, promotion of diversification, and compensation for production difficulties in areas with specific natural constraints (in order to fight against the risk of land abandonment).

The progressive broadening of CAP’s original focus and its reshaping through many processes of reform have resulted in a very large legislative framework, issued by the Council or the European Commission, which combines two components of equivalent importance: *expenditure* and *regulatory measures* related to many different areas of interest for agriculture and agri-food products.

*Expenditure measures* are traditionally organised in two areas of intervention, defined as “pillars” in the recent CAP jargon. The *first pillar* represents the set of instruments aimed at supporting agricultural products markets and farmers’ incomes; it is generally considered the core of CAP due to the size of its financial envelope; the *second pillar* is aimed at supporting the structural strengthening of the agricultural sector. The budget for the related expenditures is covered by two funds: the European Agricultural Guarantee Fund (EAGF), which provides financial resources for the first pillar, and the European Agricultural Fund for Rural Development (EAFRD), which finances the second pillar, implemented through national/regional programmes, which require financial participation by MSs (co-financing). It is worth to underline that the financial envelope for the second pillar has been traditionally smaller than those for the first one.

*The corpus of regulatory measures* traditionally includes provisions concerning marketing and producer organisations, trade with third countries and competition rules. These measures define a complex framework which represents a characterising part of CAP; but nevertheless, this policy is frequently identified only with its expenditure measures.

According the evolution of objectives, after about 30 years of substantial stability, also the general structure of the CAP and the consequent functioning of its two pillars underwent substantial changes, driven by many internal and external factors which acted in the last decade of the twentieth century (Ritson and Harvey 1991; De Filippis and Salvatici 1991; Swinnen 2011; Daugbjerg and Swinbank 2011). On the internal side, it is worth mentioning the stricter budget constraints decided with European Single Act (1987) and the resulting necessity of containing the raising costs of CAP, the creation of a single market and the following pressures towards a monetary union, the weak orientation to the market of agricultural production, the growing attention to the environmental matters and the increasing demand for food quality and safety. On the external side, the progressive of European enlargement process and the growing difficulties in making CAP support compliant with the more restrictive international commitments deriving from the GATT’s Uruguay Round come to an end in 1994 with Marrakesh agreement and the foundation of WTO. The milestones of this new phase of CAP are the Mc Sharry’s reform (1992), quickly followed by the further deepening of Agenda 2000 reform (1999) and then the Fischler’s reform (2003) which finally shaped the new institutional framework of CAP.

Concerning the first pillar, during the 1990s, the first step was taken toward shifting away from the traditional policies of market support, implemented via aid schemes based on guaranteed prices, to a more complex mechanism of support addressed directly and indirectly to producers. Farmers operating in most commodity markets (cereals, oil seeds, livestock etc.) were given a scheme of direct payment linked to the hectares of cultivated areas, calculated on the base of historical levels of production, integrated with intervention tools in case of a serious market crisis. Deepening this approach, Fischler’s reform (2003) established further cuts in the linkage between subsidies and production, strengthening the process of decoupling financial support from agricultural production in favour of a direct linkage with farmers’ status and behaviour in order to better guarantee the achievement of the above-mentioned goals (Anania and Pupo D’Andrea 2015). On the other hand, for farmers operating in specific sectors (especially Mediterranean products, including wine) the aid schemes progressively evolved toward specific sets of measures, mainly intended to support the market performance of involved actors.

Concerning the second pillar, after 1992, resources were no longer destined exclusively to stimulate the technological and structural improvement of farms; they were also directed to improve the efficiency of supply chains and strengthen the social fabric of rural communities.

Since the last CAP reform of 2013, which is currently in force, the two typical lines of CAP intervention, expenditure and regulatory measures, are implemented through three main regulations^5^ which together create a policy structured on Direct Payments (Reg. (EU) 1307/2013), Single Common Market Organisation (Reg. (EU) 1308/2013) and Rural Development (Reg. (EU) 1305/2013). Such regulations operate over the current financial period 2014–2020; their content and financial aspects are summarised in Table 1. Moreover, specific rules concerning the mechanism of distribution and use of financial resources are set by the Regulation (EU) 1306/2013.

**Table 1:**
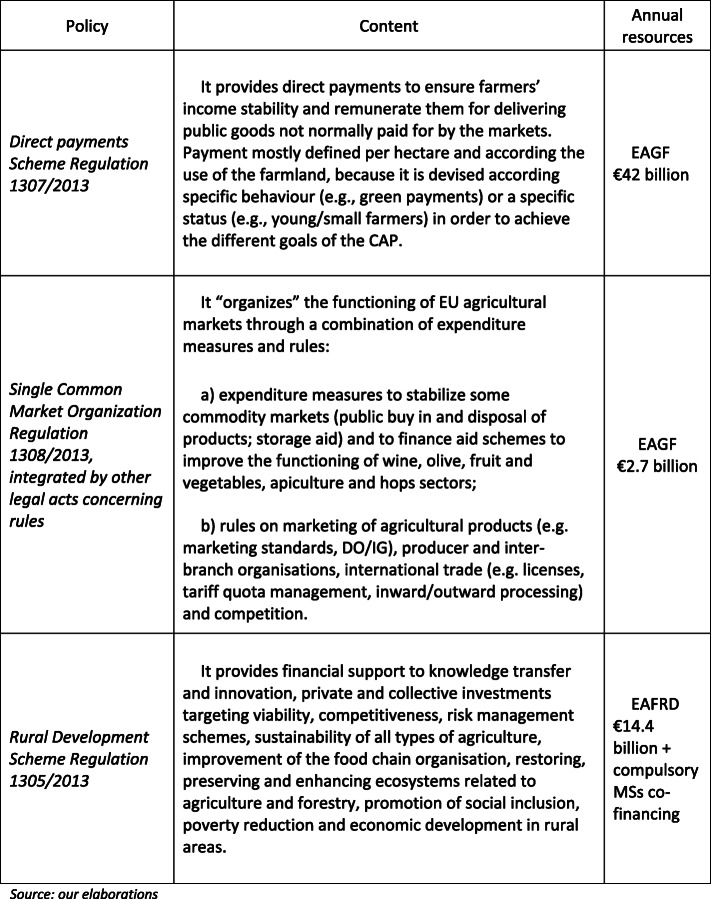
Current CAP synopsis (2014–2020)

From the point of view of rules regulating markets and producer behaviour, the CAP is complemented by many other legal acts. Just as an example, it is worth quoting the regulation concerning the use of geographical indication and designation of origin in non-wine foodstuffs (Regulation (EU) 1151/2012), the regulation concerning organic production (Regulation (EU) 848/2018) and the directive concerning the sustainable use of pesticides (Directive 2009/128/EC).

Finally, it must be highlighted that the different actions have been conceived as a part of a global policy that should operate in complementarity for the achievement of the identified objectives of the agricultural sector. However, the CAP assigns the task of ensuring this complementarity to MSs, establishing a certain degree of flexibility in the implementation rules of the tools within the two pillars.

Within CAP, the European wine policy has also been affected by a major evolution in the pursued objectives and measures implemented for their achievement. As early as 1962, the first step of wine policy was the issuing of a regulation aimed at a simple goal of information collection and unifying some preliminary aspects of legislations about wines with a declared geographical origin from the different MSs producers. At the beginning of the 1970s, many other regulatory aspects were introduced on the basis of the need to stimulate a large traditional sector towards a rapid adaptation to the dramatic changes facing by the wine market^6^: rules on viticulture, rules on wine production, definition of different types of wines, rules on labelling, introduction of guide prices for market intervention, distillation and so on (Meloni and Swinnen 2013). As a consequence, the wine policy immediately differed from that of other sectors (Scoppola and Zezza 1997). Its peculiarity within the CAP framework, as a distinctive characteristic, has been maintained during the subsequent sectoral reforms, which, among other measures, introduced a ban on new planting, a mechanism of subsidies for permanent abandonment, and financial support for restructuring and conversion of vineyards.

In the more recent period, after the failure of an attempt of radical reform in 1994 (Montaigne 1997), wine policy took part of Agenda 2000 reform process with the introduction of new tools aiming to quality improvements of wine supply, in order to better respond to demand evolution and deal with the increasing pressure of New World competitors. This process came to a full ripening with the 2008 reform that completely renewed the CAP interventions for wine, establishing a peculiar aid scheme where, for the first time, traditional market measures were combined with structural measures financed by a national envelope and substantial normative innovations (Pomarici and Sardone 2009).

This original approach was conceived to support the achievement of a set of objectives consistent with the new challenges emerging in the wine market and the commitment of the wine sector to larger social and environmental issues:

- Increasing the competitiveness of EU wine producers;
- Strengthening the reputation for quality of EU wine as the best in the world;
- Recovering old markets and winning new ones in the European Union and worldwide;
- Creating a wine regime that operates through clear, simple and effective rules that balance supply and demand;
- Creating a wine regime that preserves the best traditions of EU wine production, reinforcing the social fabric of many rural areas and ensuring that all production respects the environment.

These objectives also seem consistent and, to some extent, anticipatory of the 2013 CAP reform objectives. Indeed, as highlighted in Table 2, it is quite simple to verify that the above-mentioned goals of the improvement of the position, competitiveness and reputation of European wine in the global market, and the achievement of better market control, are perfectly coherent with the concept of viable production, as mentioned above. In the same way, the emphasis on the socio-environmental role of viticulture seems consistent with the aim of promoting the sustainable management of natural resources and, partially, with the promotion of balanced territorial development.

**Table 2:**
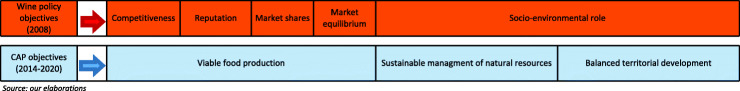
CAP and wine policy objectives: a comparison

The consistency between 2008 EU wine policy reform and the 2013 general CAP reform made it easy to include the wine policy model crafted in 2008 in the so called Single Common Market Organisation (CMO) currently in force under the Regulation (EU) 1308/2013^7^. The next section presents in detail the features of the financial and normative provisions for the EU wine sector.

## The current support to the wine sector within the CAP

Wine policy in the period 2014–2020 stems from the three main CAP regulations and is completed by other legal acts that establish operational details for their application: (i) since 2013, farmers with vineyards may benefit from income support (optional for MSs); (ii) wine sector activity is affected by market measures that include financial support and a complex system of regulatory measures for wine production and marketing; (iii) actors in the wine sector may also apply for the resources of the Rural development policy, competing with actors of other agricultural sectors in the framework of rural development plans (RDP).

### The hard core of the wine policy: wine in the single CMO

Regulation (EU) 1308/2013 lays down important prescriptions concerning wine, in terms of both expenditure measures and regulatory measures.

MSs involved in wine production have to manage the aid scheme specifically intended for wine through a 5-year National Support Programme (NSP)^8^ selecting among a set of eight possible expenditure measures, to meet the needs of their regional bodies, taking their peculiarities into account. MS are responsible for the implementation of such programmes. The expenditure measures eligible for the NSP are of different types (Table 3): five are structural (structural measures) and three target the prevention of farm revenue drops (conjunctional measures). NSP financing relies on a significant pre-assigned financial envelope, equivalent to 2.3% of the first pillar expenditures through the EAGF, corresponding to little more than one billion euro per year.

**Table 3:** National Support Programme measures: nature, objective, content and beneficiaries

Measures	Objective and content	Beneficiaries
*Structural measures*		
Promotion	For expanding the presence in the market, it is granted a support for the implementation of campaigns concerning promotion of EU wines in third countries and information about responsible consumption and UE systems of PDO/GPI in MS	Professional organisations, wine producer organisations, associations of wine producer organisations, temporary or permanent associations of two or more producers, interbranch organisations or, where a Member State decides so, bodies governed by public, private companies
Restructuring and Conversion of vineyards	For increasing quality, cost effectiveness and sustainability of grape production, it is granted a support for (a) varietal conversion, (b) relocation of vineyards, (c) replanting for health or phytosanitary reasons, (d) improvements to vineyard management techniques	Wine growers (*)
Investments	For increasing the performance and sustainability of wine making and distribution, it is granted a support for tangible and intangible investments in processing facilities, winery infrastructure and marketing structures	Wine enterprises (***), wine producer organisations, associations of two or more producers or interbranch organisations
Innovation in the wine sector	For stimulate innovations in a perspective of sustainability, it is granted a support for tangible or intangible investments aimed at the development of new products, processes and technologies	Wine enterprises (***), wine producer organisations and temporary or permanent associations of two or more producers. R&D centres may participate in the operation pursued by the beneficiaries. IBO may be associated to the operation
By-product distillation	For ensuring quality of wine, while protecting the environment, it is granted a support for voluntary/obligatory distillation of by-products of wine making	Distillers of by-products of wine making
*Conjunctional measures*		
Mutual funds	For stimulation the adoption of tools against market fluctuations, it is granted a support for the setting up of mutual funds	Wine growers (*) or wine producers (**)
Harvest insurance	For safeguarding producers’ incomes in case of natural disasters, adverse biotic/abiotic events, it is granted a support to the subscription of insurance contracts	Wine growers (*)
Green harvesting	For preventing market crises and compensating the loss of revenues, it is granted a support in favour of the removal of grapes in immature stage	Wine growers (*)

Source: Regulation (UE) 1308/2013 and Delegated Regulation (EU) 1149/2016

*‘Wine grower’ shall mean a natural or legal person, or a group of natural or legal persons, whatever legal status is granted to the group and its members by national law, whose holding is situated within Community territory, as defined in Article 299 of the Treaty, and who farms an area planted with vines (Art. 2 of Regulation (EC) 436/2009)

**Producers of the products referred to in Part II of Annex VII to Regulation (EU) 1308/2013 different from wine growers

***Wine enterprises producing or marketing the products referred to in Part II of Annex VII of Regulation (EU) 1308/2013

The five NSP measures of a structural nature were conceived to strengthen the competitiveness of the wine sector in MSs, allowing the financial support of improvements at different level of the supply chain; the flexibility accorded to MSs concerning the resources destined to the single measures allows the intervention to be adjusted according to specific needs.

The measure for “restructuring and conversion of vineyards” is intended to contribute to the competitiveness of the EU wine sector offering opportunities to renovate the operative conditions at the stage of the wine production chain that generates the largest share of wine production costs and the highest environmental impact as consequence of the heavy use of fungicides; this represents the origin of the value created by wine production. By including this measure in the NSP, wine growers are put in a position to improve their sustainability not only by changing their vineyard management techniques but also by choosing better sites and varieties more suited to the eco-physiological condition of the farm.

The “investments”, “innovation” and “by-products distillation” measures are to support wine quality from the perspective of sustainability. The “investments” and “innovation” measures support technical improvement and innovation in wine making and wine distribution to improve the value of products (better intrinsic and extrinsic characteristics, optimised costs) and produce a positive effect in terms of energy savings, global energy efficiency and environmentally sustainable processes. The “by-product distillation” measure intends to prevent the quality-damaging practice of excessive exploitation of wine pomace and support the environmentally friendly disposal of wine making by-products.

Finally, the measure “promotion” is intended to support the presence of EU wine in the global market. To comply with the opposition of the Directorate General for Health and Consumer Protection of the European Commission (DG SANCO) to policies that could result in an increase in alcohol consumption in the EU, the measure lays down different prescriptions for action inside and outside EU; as a matter of fact, only outside the EU it is possible to implement true promotion activities based on public relations, promotion or advertisement measures, and participation at events of international importance; such activities should rely on market studies and structured plans, and their effects must be ex post evaluated.

**Fig. 1:**
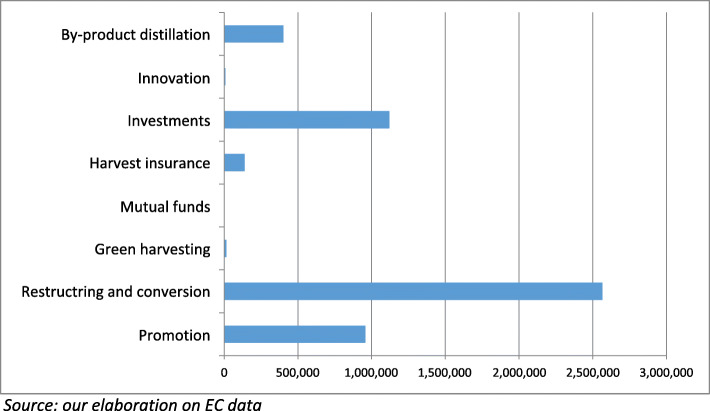
Wine NSPs financial execution and programming: 2014–2018 (000 euros)

As shown in Fig. 1, the structural measures, with the exception of innovation, are the most financed by MSs. What is remarkable is the concentration of resources on the restructuring and conversion of vineyards, which account for more of the half of the budget of the aid scheme for the wine sector. The measure “innovation”, instead, has virtually never been included in NSPs. This cannot be considered the result of an underestimation of the importance of innovation in the wine sector but, likely, the result of a strategic decision in MS to address wine enterprises to the other sources of innovation supporting funds. Concerning the measure promotion almost the total expense is destined to action for third countries. While informative actions concerning European wine classification addressed to EU markets has been very limited and the information for responsible consumption virtually absent (Agrosynergie GEIE 2018).

The NSP *measures of conjunctural nature*, “harvest insurance”, “mutual funds” and “green harvesting”, offer a set of tools to assist single enterprises in facing different economic risks. These are conceived as preventive instruments able to encourage a responsible approach to crisis situations after the dismantling of the traditional market protecting measures (price support, distillations, private storage, must aid) in force until 2008. The green harvesting prevents losses deriving from insufficient demand for farm products at the time of full grape ripening and is consistent with sustainability principles, as it avoids the production of wine that could be destined to downgrading via distillation. Mutual funds and insurances compensate producers in case of, respectively, revenue losses due to market crisis or yield reduction for biotic or abiotic reasons, which are more and more frequent due to climate change. As shown in Fig. 1, insurances received very little in NSPs, while mutual funds were never included in it, despite being technically feasible and financially sustainable (Trestini et al. 2017). Even though both mutual funds and insurances are consistent with the EU policy orientation toward a larger diffusion of risk management practices in EU agriculture. As a matter of fact, all MSs took advantage of the reasonable equilibrium between supply and demand in the wine market to concentrate NSPs resources on fostering competitiveness. However, these tools still present a limited implementation in all other sectors (European Commission 2017b).

The NSP tools targeting market and revenue stabilisation may be reinforced by optional national payments for distillation of wine in cases of local crisis; MSs may make national payments to wine producers for the voluntary or mandatory distillation of wine in justified cases. The overall amount of payments available in any given year shall not exceed 15% of the globally available funds per MS.

Interestingly, as shown in Table 3, beneficiaries of support measures are not just wine growers; three measures only are exclusively addressed only to these actors, i.e. who farm an area planted with vines, and the others have, as beneficiaries, a larger array of actors involved in wine production or marketing. As a result of this particular characteristic, the intervention in the wine sector can be considered the only true vertical policy within CAP. The inclusion among spending-measure beneficiaries of not-strictly-agricultural actors is due to the structure of the European wine industry and the nature of wine grapes. Wine grapes assume value only as input of the wine production process, but their perishability gives them a very limited window of exploitation in space and time; therefore, to guarantee value to grapes, it is necessary to guarantee the existence of a valid processing activity (wine making and bottling). These production phases are not always carried out on the farm, emphasising the important role of non-agricultural actors; this is particularly true if they are located in or near the grape growing areas, especially with regards to the production of wines identified with their geographical origin^9^ (European Parliament 2017; Anderson and Pinilla 2018; Alonso et al. 2018).

The regulatory measures concerning EU wine defined within the single CMO are also very important; they cover many aspects of production and marketing of grapes and wine, and to a large extent, these measures are essential to control the quality level of EU wines and ensure market stability.

Grape processing to make wine may be conducted only with explicitly permitted practices and technological solutions (taking into account oenological practices recommended by the International Organisation of Vine and Wine - OIV)^10^, and grape varieties admitted for wine production should be authorised by MSs.

General rules are defined with regard to the labelling and presentation of EU wines and particular rules are provided concerning the identification and presentation of EU wines using geographical names. Following a consolidated tradition, EU protects the authorised geographical names^11^, recognising the property rights of concerned producers. According the World Intellectual Property Organization (WIPO) approach, these geographical names may pertain to two different categories, the designation of origin and the geographical indication, which refers to a different relationship between the sensory profile of wine and the area where it is produced^12^. Therefore, EU wines with a recognised geographical origin are presented to consumers as Protected Designation of Origin (PDO) or as Protected Geographical Indication (PGI).

Moreover, to gather information to monitor the wine market and ensure the traceability of wine products, MSs are committed to communicating to the Commission an Inventory of their production potential, based on the vineyard register; rules are defined concerning compulsory declarations, documents accompanying consignments of wine products and the wine sector registers to be kept.

As in the case of other agricultural products, the single CMO assigns a relevant role to the integration of wine actors (European Commission 2016b). Producer organisations (PO) and their associations are recognised in the wine sector as a strategic instrument in concentrating supply, improving marketing, planning and adjusting production, promoting best practices and providing technical assistance for their members. The interbranch organisations (IBO) are considered important in allowing dialogue between actors in the supply chain, and in promoting best practices and market transparency; in particular, they may establish marketing rules to regulate supply^13^. However, POs and IBOs in the wine sector are not directly involved in the implementation and management of the NSP, in contrast to the implementation and management of aid schemes in the other concerned sectors; in the wine sector, POs and IBOs may only be beneficiaries of some NSP measures (see Table 3). In the wine sector, IBOs have a long tradition in the protection and promotion of wines with recognised geographical origin, despite the use of different organisation models in different MSs^14^. Less widespread are, on the contrary, POs; this likely depends on the peculiar structure of the wine industry, which is characterised by a pervasive diffusion of cooperatives and by the absence of financial incentives that, on the contrary, has been granted in other sectors. Anyway, as already indicated in Table 2, POs and IBOs may apply for some measures of NSP.

Finally, Regulation (UE) 1308/2013 includes a new scheme governing the area under vine (production potential), which is now based on a system of planting authorizations implemented for the period 2016–2030. The persistence of a control mechanism of production potential represents the most idiosyncratic characteristic of the wine policy, which no longer has an equivalent in other sectors regulated by CAP. This ban has also represented the most discussed items of the last process of revision and is the result of a compromise between two opposite positions: on one side, the EU Commission that was strongly oriented towards a total liberalisation of the sector; on the other, the traditional producing countries, mostly worried about the risks of new market unbalances and of the financial impacts of a different regime on the value of vineyards (European Parliament 2012). So, with the aim of ensuring the orderly growth of production potential, a scheme has been introduced for new vine plantations based on administrative authorisations granted to beneficiaries without a cost, distributed in a non-discriminatory manner but with specific rules defined by MSs (European Commission 2019b)^15^. The new scheme permits a yearly maximum 1% increase in the area under vine according to the national inventories, respecting well identified conditions. In order to pursue specific objectives, MSs may implement the scheme paying particular attention in favour of specific areas or types of products, the contribution to environmental protection, young farmers, improvement of competitiveness, etc. (Sardone 2016).

### Direct payment and rural development schemes: opportunities and critical issues for wine

#### Wine and income support (direct payments)

The current income support policy (Regulation (EU) 1307/2013) allows MSs to include vineyards among the areas eligible for the direct payments. The option to include vineyards for direct payments has been adopted by most EU wine producing countries: Italy, France (only in some areas), Spain, Greece, Belgium, Croatia, Malta, Netherlands, Malta and Luxemburg. The calculation of direct payments for areas under vine considers that vineyards, like all permanent crops, are considered “green by definition” and are automatically allowed to receive the additional payments for agricultural practices beneficial to the climate and environment (green payment). The payment per hectare is very different in each country, as a consequence of heterogeneous national decisions concerning computation rules. Unfortunately, the total size of payments to wine growers in each country is not available.

#### Wine and the rural development measures

Actors in the wine sector may apply for financial support from the rural development policy (Regulation (EU) 1305/2013), competing with actors belonging to other agricultural sectors, as no pre-assigned budget for grape or wine producers exists^16^. In each MS, actors’ access to financial support in the framework of the rural development scheme is planned by rural development plans (RDP) defined at the national and/or regional level. Such plans are set up by selecting among a set of 20 measures and allocating to each selected measure a share of the available budget, respecting some constraints; plan settings have to be based on a SWOT analysis of the country/region concerned, and plan projects are approved by the European Commission. The available measures cover many fields of agriculture, forestry and more generally social and natural environmental protection. Grape and wine producers may be primarily interested in specific measures concerning support for new participation in quality schemes, the setting up of producers groups and organisations, investments in physical assets, business start-up (especially by young farmers), adoption of particular agri-environment-climate commitments or organic farming, creation of clusters, and networks to establish and run the operational groups of the European Innovation Partnership for agricultural productivity and sustainability (EIP).

It is possible to observe that some of the measures available for the NSP, both structural and conjunctural, may also be selected for RDP. This determines a potential overlap of support tools with the risk that the same operation (for example, the purchase of equipment) is funded twice, in conflict with the EU rules. Therefore, in each MS, or region therein, it has been necessary to decide which operations to support through the NSP and which through the RDP (the so-called problem of demarcation). Almost all MSs decided to finance the renovation of vineyards only through the NSP despite the RDP (measure for physical assets). The investments in the technical improvement of wine making and distribution have been, instead, supported in part through the NSP and in part through the RDP, while innovation has been mostly supported by the RDP, in the framework of EIP. Anyway, as in the case of direct payment, the share of rural development resources destined for the wine sector is not declared by the European Commission.

### EU policy for wine: tools vs. objectives

The EU wine policy in a broad sense, as shown in the previous section, reflects, although with a specific identity, the shape of CAP. However, this complex set of financial and regulatory tools does not pursue all of its objectives in a balanced way.

With a pure qualitative approach, Table 4 highlights the correspondence between wine policy tools and CAP objectives and sheds light on at least three relevant issues.

**Table 4:** Relationship between CAP objectives and measures of wine policy (2014–2020)

	CAP’s objectives (2014–2020)		
	Viable food production	Sustainable management of natural resources	Balanced territorial development
***Single CMO (Reg. 1308/2013)***			
***a - measures of support: NSPs***			
Promotion	**X**		
Restructuring and conversion of vineyards	**X**	**X**	
Green harvesting	**X**		
Mutual funds	**X**		
Harvest insurance	**X**		
Investments	**X**	**X**	
Innovation in the wine sector	**X**	**X**	
By-product distillation	**X**	**X**	
***b - regulatory measures***			
Oenological practices and rules about viticulture	**X**		
PDO and PGI	**X**		
Labelling (varietal wines)	**X**		
PO and interbranch org.	**X**	**X**	
Declarations and communications	**X**		
***c - scheme of authorisations for vine planting***	**X**	**X**	**X**
***Direct payments (Reg. 1307/2013)***	**X**	**X**	**X**
***Rural development measures (Reg. 1305/2013)***	**X**	**X**	**X**

Source: our elaborations

First of all, all measures target the viable food production objective. Indeed, the goal of viable food production can be considered the focal point of wine policy, intended as specific wine spending and regulatory measures, although it must be underlined that measures aimed at income stabilisation still appear, as already shown in Fig. 1, to be characterised by limited implementation and by minor financial importance.

Fewer measures target the sustainable management of natural resources. Nevertheless, all measures related to the efficiency of the production process do include sustainability and, in particular, improvements to environmental sustainability; moreover, wine actors may apply for measures in the rural development policy that directly target environmental goals (but, as mentioned, they do not enjoy a pre-assigned budget) and have to comply with some standards on good agricultural and environmental conditions of land (cross compliance principle) to receive the full amount of direct payments.

Balanced territorial development, intended in the sense of resilience of vitivinicultural areas to significant specific changes of their position in wine market, appears only marginally targeted by the core of EU wine policy, as this objective could be directly pursued by only giving priority to disadvantaged areas in distributing the authorisation for new planting, and room can be found in vineyard eligibility for direct payments (where provided). As a matter of fact, it is the rural development scheme that may offer effective tools to pursue the objective of balanced territorial development if the possibility offered by the Regulation (EU) 1305/2013 is used to implement specific territorial programmes.

Unfortunately, it is difficult to add to the qualitative analysis a quantitative evaluation of resources destined to each objective because, first, the total amount of EU resources destined to wine is not declared in EU accounting reports and, second, many measures, as highlighted in Table 3, have multiple objectives, making it difficult to estimate the shares of resources delivered to the wine sector that are actually destined to each objective.

The financial resources destined to wine through NSP are known but, as previously mentioned, the EU accounting procedures do not allow us to know the share of resources destined to horizontal measures (direct payments and rural development) delivered to single sectors.

In the absence of a feasible quantification based on the European Commission reports, it is possible to refer to the (Anderson and Jensen study 2016) which, integrating OECD computations of agriculture support indicators with additional elements, have estimated that EU wine producers received, in the period 2007–2012, an average of about €2.3 billion per year, corresponding to approximately €700 per hectare or €0.15 per litre of wine produced^17^. About the half of this estimated support is directly delivered by NSPs and the rest comes from customs tariffs, EU and national resources of rural development plans, and resources indirectly destined to wine producers supporting education, public R&D activities and infrastructure^18^.

Considering that more than three fourths of resources delivered by NSPs (those destined to vineyard restructuring, investments and by-product distillation)^19^ are functional to the achievement of results concerning viability of production and management of natural resources and that measures of RDPs are also mostly addressed to viability of production and management of natural resources, it is possible to conclude that these two policy objectives together are the primary target of financial resources destined to the wine sector. On the other hand, there is no evidence of substantial financial support for balanced territorial development.

## The performance of EU wine policy: an analytical evaluation

Before analysing the reform proposal under discussion, it is useful to understand how the EU wine sector is dealing with the three big challenges of the current CAP—production viability, natural resources management and balanced territorial development—which, as better explained in the next section, are substantially taken up by the proposed new CAP as well. The objective of the analysis is not to evaluate the effectiveness of the wine policy, as the performance of the sector is the result of many factors and the policy is just one among them. Among the many factors affecting directly and indirectly the sector over the considered period, it is worth to mention, related to the macroeconomic conditions, the introduction of the euro currency and changes in real exchange rates, the financial crisis started in 2007, the accelerated development of new geographical areas, the WTO impasse and the growing search of bilateral agreements, the strong attention for environmental and climatic issues (Mariani et al. 2014b; Pomarici 2016; Anderson and Pinilla 2018). On the side of wine flows, the considered period is characterised by a substantial stability of global supply, despite with some relevant harvest fluctuations (remarkable the large harvest in 2004, see Fig. 3), a steady growth of global consumption up to 2008, followed by a decrease and an incomplete recovery, and a substantial increase of international trade characterised by the following: changes in the composition of flows, with an increase of sparkling wine share and a modernisation of bulk wine; new trade routes, with an increasing role of re-exporters; redefinition of the competitive scenario with the resistance of European exporters to the catch-up process of producing countries in the New World and an increased complexity of the market’s regulatory framework (Mariani et al. 2012; Anderson and Wittwer 2013; Morrison and Rabellotti 2016; Anderson and Pinilla 2018).

The objective of the analysis is to identify the critical issues that the reform must address and against which to evaluate its potential effectiveness. To this end, a set of synthetic indicators available for all EU wine producing countries and some useful available reports, mainly elaborated by the EU, are considered. In particular, the analysis covers the new century, updated with the most recent available data, focusing on trends from 2009, which represents de facto the starting point of the current wine policy that, as specified in the previous section, has only been marginally modified in 2013 to better address the three main CAP objectives.

### Production viability

Production viability is evaluated as the result of three dimensions: market performance of supply, profitability of production and market equilibrium.

To evaluate *market performance* of EU wine supply, a set of indicators was selected that refers to the main aspects of the positioning of EU wine producers in the global market: area under vine, volume of production, value of production in comparison with the overall agricultural sector, international trade.

**Fig. 2:**
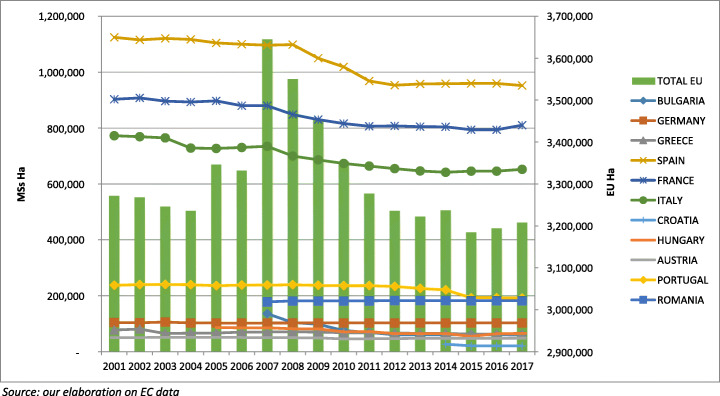
EU production potential by MS (2001–2018)

Figures on area under vine (production potential) show a quite complex evolution (Fig. 2). The aggregate EU area under vine grew moderately between 2001 and 2009, but this increase is just a consequence of the entrance of new wine-producing MSs (2004 and 2007). Indeed, focusing on the total area under vine in Mediterranean producing countries, the trends appear to be drastic decline. After 2009, with the contribution of the EU programme of permanent abandonment launched for a limited period (until 2011), the effect for the entire EU area under vine was a reduction of 175,000 hectares in 3 years (5% of the EU vineyards). More recently, implementation of the new rules on production potential (annual growth up to 1% of the planted vineyards per MS) plus the last years of management of old planting rights held in portfolios by wine producers (abolished at the end of 2015) have contrasted the previous trend^20^. So, the overall effect has been an increase in total EU vineyards, despite different trends between MSs, with a significant increase in land planted in Italy and France and a relevant statistical effect produced by Croatia, starting in 2014. In general terms, the two reforms of 2008 and 2013 have determined, first of all, an acceleration in the process of decline, then a gradual recovery, but limited to some producing countries. The result has been a small decrease in the EU share in the world area under vine over the first decade of this century and then its stabilisation in more recent years. The aggregate evolution within each producing country is the result of the combination of different regional trends, as better explained later discussing fulfilment of the balanced territorial development objective (the “Balanced territorial development” section).

**Fig. 3:**
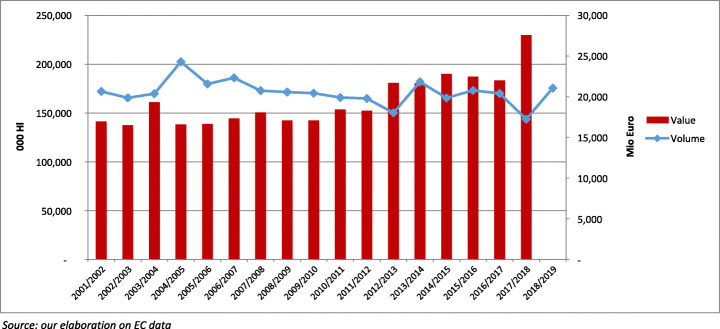
EU wine’s production: volume and value (2001–2018)

EU wine production has evolved since 2001 with a path similar to the world trend. After 2009, also as consequence of the elimination of distillation facilities and the drastic reduction in areas under vine, production decreased, but the share of EU in world production remained substantially unchanged, wavering between 62 and 65%. Almost all larger producers, except in Spain, contributed to the production reduction after 2009. More specifically, Italian production almost constantly decreased, and France was characterised by a very irregular trend. In the last 5 years, almost all MSs have experienced a very high variation in production volume due to the influence of the evolution of area under vine and more so the weather or vineyard health conditions (Fig. 3). These production trends have almost systematically been converted into corresponding internal price variations, the tendencies of which show significant differences by wine type and MS, with a generally higher volatility for wines without varietal or geographical specification. Concerning these latter, it is possible to observe a strengthening of share represented by PGI and PDO wines, in comparison with generic wines, the volume of which corresponds on the EU average to around 60% of overall production (2009–2018). In the more recent period, PDO and PGI wines have demonstrated a fair capacity to strengthen their market position, through an improvement of average prices in the global arena. The overall effects of these joint evolutions can be verified with an analysis of figures about the trend in the value of wine production measured at farm level (Eurostat). After 2009, despite the decrease in volume and thanks to the expansion of PGI and PDO wines, the value of production (at constant prices) increased annually by 5% (compound annual growth rate—CAGR) and the share of this value on total agricultural production remained stable at around 5%. Anyway, the differences among MSs must be underlined, with shares that vary from 11 to 13% in Italy, France and Portugal, to around 2 to 3% in Germany and Spain. Despite the national differences, the global effect of this joint evolution results in a generalised and sensible increase in the output value per hectare, which grew by 11%.

**Fig. 4:**
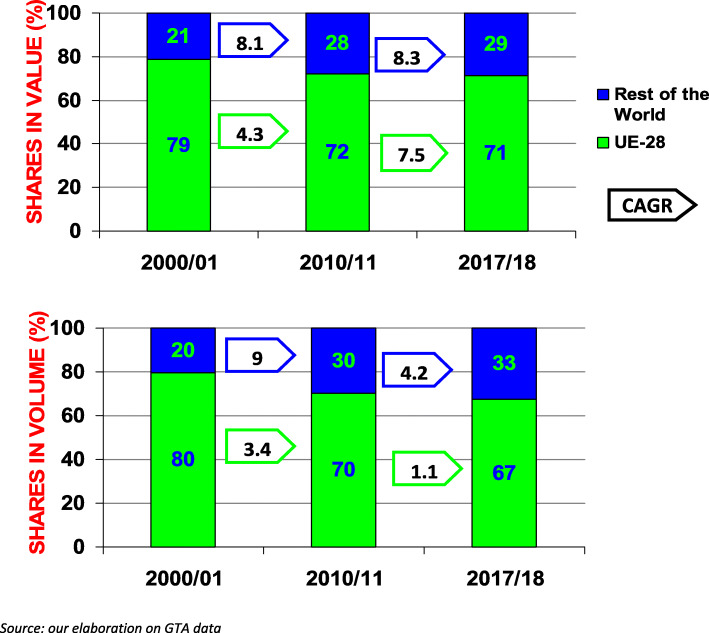
EU and rest of the world export shares and growth rate (value and volume 2000/2001–2017/2018)

Exports by EU wine producers have grown continuously in value and volume from 2000 to 2018; after 2009, the growth occurred at a lower rate but with a sensible increase in unit value (from 2.4 to 3 between 2010 and 2018) (Fig. 4). Over the considered period, the positive performance of producers in non-EU countries must be underlined, and they expanded their exports at a higher rate than EU producers. Already before 2009, the non-EU export CAGRs were considerably higher with respect to the EU; after 2009, the CAGR in value become just a little higher than that of the EU but was four times the EU’s in volume. The result is a continuous reduction in the EU share of world exports in terms of volume (from 70 to 67%) and in value (72 to 71%). These results evidence a lack of capacity to maintain or gain market positions in the global arena or to seize the opportunities stemming from expanding world demand as well competitors have been. In the most recent period, among big European producers, Spain shows the relatively best results, increasing its share in volume, with the share in value remaining stable. France reduces its share in volume and value, while Italy is characterised by a reduction in volume associated with a comparatively higher increase in value of wine sales in the global market.

Evaluation of the second dimension of viability, the *profitability of production*, takes into account the complex structure of the wine industry, using indicators from two different sources: indicators derived from the Farm Accountancy Data Network (FADN)^21^ and those from balance sheet databases. FADN collects information about all MSs with regard to production, structural characteristics, financial situation and income of European farms, organised by technical specialisation. FADN therefore allows us to focus on the economic performance of wine growers, who are mainly small actors, frequently not directly connected with the final market, and sell grapes or wine on intermediate markets. Balance sheet databases collect economic data of companies, which are usually at least relatively large. Such databases therefore allow us to focus on the economic performance of medium/large wine enterprises, sometimes run as agricultural companies.

**Fig. 5:**
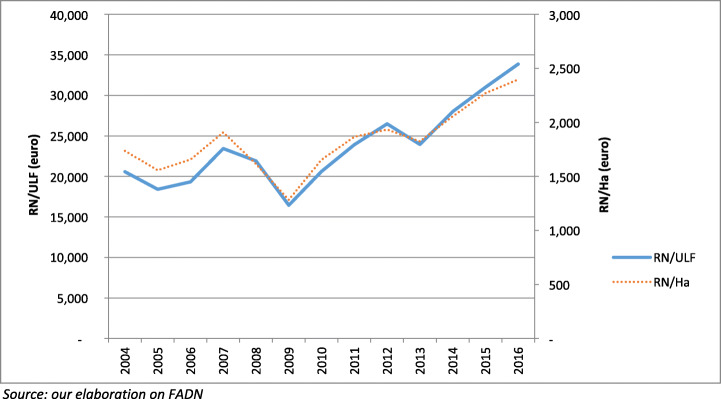
EU farms specialised in wine growing: average farm results (2004–2016)

Analysis of FADN data, first of all, reveals that farms oriented toward grape and wine production, on average, are among the most profitable, with a limited number of exceptions. Moreover, analysis of evolution of net revenue per hectare (NR/Ha) and per unit of familiar labour (NR/UFL) shows, in particular between 2009 and 2016 (last available data), a sensible increase, especially for the latter (Fig. 5). In addition, looking at data about the entire EU, the minimum value between 2010 and 2016 is higher than the average of the pre-reform period (2004–2009). A closer observation of the same indicators in the largest producers (France, Italy, Spain, Germany) evidences more variability, though the general progressive improvement in values remains confirmed.

The evaluation of the profitability of larger wine enterprises is more complex, as a homogeneous EU source is lacking; therefore, it is necessary to rely on different sources of national information, supplying data elaborated with different methods. Data on the ratio between earnings before interest, tax, depreciation and amortisation (EBITDA) and sales (EBITDA Margin) in recent years (2011–2015) reveal that the profitability was fair in Italy (between 5 and 7%; Mediobanca 2018) and Spain (between 4 and 7%; INE 2016), while it was rather high in France (between 9 and 10%; Credit Agricole 2016).

**Fig. 6:**
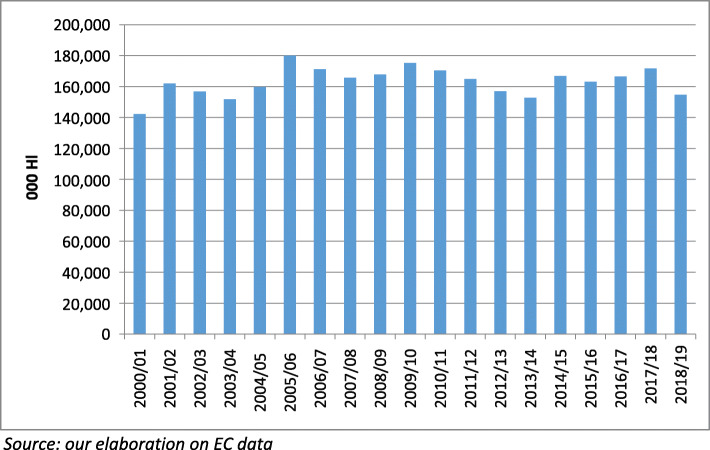
EU opening stocks by vintage year: 000 Hl (2000–2018)

Finally, in relation to *market equilibrium*, the performance of wine sector can be evaluated through a less extensive set of information. The most relevant is represented by the evolution of data about wine stocks, which reveals a progressive stabilisation for all wine producing MSs after 2009, while the previous years were characterised by a general increase (Fig. 6). Certainly, the mentioned area under vine reduction and some poor harvests contributed to stock stabilisation. Regarding the composition by wine type, a more significant variability must be underlined in stocks for wine without any identification (no PDO, PGI or varietal), which is confirmed within the different large producers of the MSs.

### Management of natural resources

Concerning *management of natural resources*, a comprehensive evaluation of the performance of the EU wine sector with respect to the reduction of environmental impact is very difficult, mainly due to lack of homogeneous updated data. The available data on the use of pesticides in the EU show that viticulture is a very intensive user in comparison with other sectors (Eurostat 2007; ISTAT 2011). According to ISTAT, more recent data for Italy demonstrate an increase in pesticides used for grape protection between 2010 and 2015: from 18.6 to 22.8 million kilogrammes of active principles, considering all types of pesticides. However, such data is difficult to interpret as current statistics on pesticide use consider simply the weight of active principles, without taking into account their toxicity and environmental impact.

Indeed, the European wine sector is remarkably sensitive to the issue of sustainability in general and the environmental impact of production in particular. This attention to environment is proven, for example, by the increase in organic viticulture and the spread of numerous initiatives related to sustainability. Between 2012 and 2017, the area under vine that was cropped using organic production rules increased in the EU by about 50%^22^. On average, the share of organic vineyards is near 11%, with the largest share in Italy (16%), while the largest area is in Spain (Table 5). Moreover, following a global trend started by the experience of the California Sustainable Winegrowing Alliance, in each European producing country, driven by ad hoc producer communities, producer organisations/syndicates, mainly cooperating with research centres, have developed initiatives concerning sustainability indicators, self-assessment procedures, production protocols, third party certification schemes with the objective to improve and certify the compliance of wine production with principles of sustainability (Szolnoki 2013; Galletto et al. 2014; Pomarici et al. 2015; Gilinsky et al. 2016; Jourjon et al. 2016; Merli et al. 2018).

**Table 5:** Area under vine converted and under conversion to organic farming in main EU producing countries, 2012–2018

	2012	2013	2014	2015	2016	2017	2018	Share on total area 2018
	(Hectares)							(%)
Spain	81,262	83,932	84,381	96,591	106,720	106,897	113,419	11.9
Italy	57,347	67,937	72,361	83,643	103,545	105,384	106,447	16.2
France	64,801	64,610	66,211	68,579	70,732	78,502	94,020	11.6
Germany	5,153	5,900	6,300	6,766	7,007	7,201	7,860	7.6
Greece	4,997	4,718	4,388	5,431	4,033	4,424	4,564	7.3
Austria	4,259	4,414	4,677	5,100	5,088	5,663	6,001	12.3
Bulgaria	2,058	3,872	2,914	4,199	5,390	4,092	3,990	6.5
Portugal	2,974	2,783	2,772	2,719	3,074	3,504	3,657	1.9
Romania	1,649	1,976	2,089	2,160	2,024	2,169	2,713	1.5
Hungary	1,206	1,219	1,198	1,325	1,637	1,716	1,759	2.7
Croatia	634	791	931	913	1,119	1,010	1,002	4.9
**UE 28**	**228,048**	**244,325**	**250,314**	**279,805**	**312,649**	**322,914**	**347,981**	**10.8**

Source: our elaboration on data Eurostat

### Balanced territorial development

The extent to which the evolution of the EU wine sector has been consistent with the achievement of *balanced territorial development* can be partially evaluated through data about the area under vine in individual regions of MS (national inventories). This reveals notable differences in all main producing countries, with many areas showing a dramatic decrease in area (Table 6). For example, considering only larger administrative units, in Italy, the Lazio, Sicilia and Puglia Regions suffer a remarkable decrease in the area under vine, in France, the Pyrénées Orientales, Aude, Gard and Hérault Departments, in Spain, the Extremadura, Valeciana, Murcia and Andalucia Autonomous Communities and, in Portugal, the Estremadura and Ribatejo. These important territorial reductions have been partially compensated by significant increases in the areas under vine of other regions, as in case of Alentejo in Portugal (Montaigne et al. 2012) or of Veneto and Friuli Venezia Giulia in Italy, characterised by a positive evolution of their production potential, mainly thanks to the internal transfers of vineyards among regions, allowed until the end of 2015 (despite some specific national restrictions), under the previous planting rights scheme (European Parliament 2012). In more recent years, the rules of the new scheme of authorisations have strongly reduced the opportunity of interregional mobility, putting in evidence the fact is more relevant the impact produce by the rules of implementation of the scheme, rather than the introduction of a ban or limitations to new vineyards (Montaigne et al. 2012).

**Table 6:** Area under vine evolution: regional differences in selected EU Member States

	Area under vine reduction (CAGR)		Administrative units with area under vine reduction double then national average in 2009/2017 (*)		
Member State	2001/2008	2009/2017	Number of adminstrative units	Their share on MS area under vine	
				2009	2017
France	-0.9%	-0.31%	24 out of 51	33%	32%
Italy	-1.4%	-0.64%	8 out of 21	33%	29%
Spain	-0.3%	-1.22%	5 out of 17	9%	7%

*: France: departments (with more then 500 ha under vine); Italy: regions; Spain: autonomous communities

Source: European Commision (2017)

Summarising, the territorial data about the evolution of areas under vine suggest that the average positive value of profitability outlined above hides important regional differences. From this perspective, it seems possible to assert that the productive structure of vitiviniculture does not look viable in all European wine producing areas and therefore the wine sector is not able to play the socio-environmental role pursued by CAP and the wine policy.

### Global evaluation

Previous analyses, despite having different degrees of soundness with respect to different issues, allow an evaluation of how the performance of the EU wine sector fulfils EU policy objectives, as defined in 2013. The result of this evaluation is summarised in Table 7.

**Table 7:**
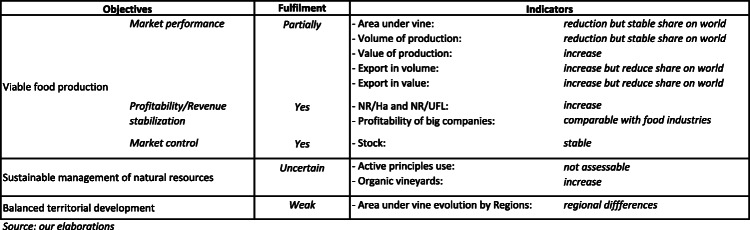
Wine sectoral performance vs. policy objectives

The evaluation of the accomplishment of viable production relies on a comprehensive and sufficiently detailed set of different indicators. Globally, this goal can be considered quite satisfactorily reached, despite the existence of some grey areas. In particular, while the conditions of market equilibrium and profitability seem to be in line with expectations, the performance of EU wine in international trade is not fully coincident with the desired results. Indeed, despite the increase in European exports, its shares, in value and volume, of world wine exports are decreasing. Likely, the slower increase in EU exports in comparison to competitors in non-EU countries, despite the incisive direct support given by the specific NSP measure (Corsinovi and Gaeta 2017), could have many reasons and in particular the lower capacity of the EU to negotiate commercial agreements (Mariani et al. 2014a).

Concerning natural resources management, the evaluation of sector performance is basically deductive. Important efforts to improve environmental sustainability of grape and wine production are evident but, as mentioned before, no updated significant quantitative indicators about the environmental performance of vitiviniculture are available. Nevertheless, referring to the wine policy, the environmental constraints (greening) defined in the CAP first pillar for the wine sector have been absolutely inadequate for stimulating a widespread awareness of environmental implications of grape production. Despite this, it must at least be recognised that the incentives to the adoption of organic production methods, supported by rural development measures, gave a considerable boost to the growth of vineyards cultivated with eco-friendly criteria. This is an important result (likely threatened by new rules about the use of copper) also because it is determining a remarkable spill-over effect: pressure on non-organic producers to adopt more sustainable production processes.

Lastly, as the data show a heterogeneous evolution of the area under vine among EU territories, with some relevant reductions in investments in vineyards in several traditional wine production areas, often included in the list of less developed areas in the EU, it seems possible to state that the achievement of a more balanced territorial development has not really been accomplished.

Evaluation of the wine supply performance clearly reveals an incomplete fulfilment of identified policy objectives. In particular, the performance is quite satisfying with respect to competitiveness of EU wine supply as a whole, while the fulfilment of the objectives of balanced territorial development and of natural resources management does not appear satisfactory. This is not considered by the European Commission as a reason to deny the worth of the general framework of the current wine policy, characterised by a mix of specific wine expenditure measures (combining first and second pillar interventions), regulatory aspects and access to horizontal measures belonging to income support and rural development policy.

After all, the Executive Summary of the recent *Evaluation of the CAP measures applicable to the wine sector* claims “The EU framework provided added value. In particular, the adaptation of the sector to market demand would have been slower without EU funding and might have further left small players behind. The EU framework was a key instrument in creating a level playing field among Member States” (Agrosynergie GEIE 2018, p. 5).

Indeed, as shown in the next section, these types of arguments have been taken into account when writing the CAP reform proposals as the current structure of wine policy is maintained, despite being within a renewed general framework.

## EU wine policy after 2020

### General features of the CAP reform: key facts

In the tricky socio-economic context of the EU, the new proposals launched in June 2018 identify an increasingly developed set of goals for CAP interventions, including the three dimensions of sustainability: economic, environmental and social. This process has resulted in the identification of nine specific objectives set at EU level, with a clear major importance assigned to needs related to environmental protection and climate change mitigation—anticipating the centrality assigned to these topics by the European Green Deal at the end of 2019—and a more evident emphasis on issues related to the capacity of agriculture to produce public goods and meet the needs of society. The 9 specific objectives of the future CAP are the following:

- Ensure a fair income to farmers;
- Increase competitiveness;
- Rebalance the power in the food chain;
- Climate change action;
- Environmental care;
- Preserve landscapes and biodiversity;
- Support generational renewal;
- Vibrant rural areas;
- Protect food quality and health.

**Fig. 7:**
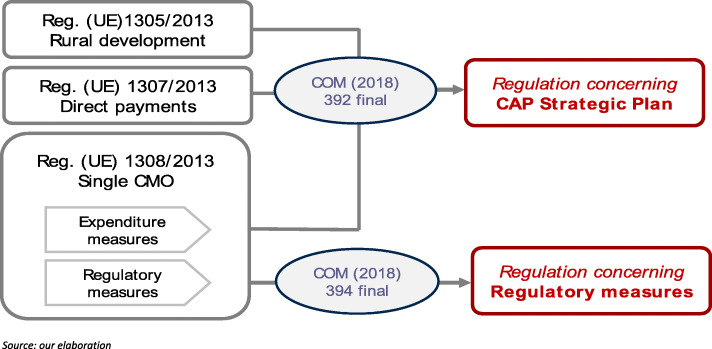
Old and proposed CAP legal act structure

From the point of view of legal acts proposed to shape the CAP after 2020, the reform process relies on two proposals of regulations (Fig. 7): the *CAP Strategic Plan Regulation* (COM/2018/392 final) and the *Amending Regulation* (COM/2018/394 final)^23^. The CAP Strategic Plan Regulation calls for major changes to the management of measures providing financial support to EU agriculture: all types of financial support—market measures, rural development measures and income support—have to be planned by the MS within a single framework, drawing up a plan called CAP Strategic Plan. The amending regulation modifies the current Regulation (EU) 1308/2013, eliminating all articles concerning spending measures and leaving, with some modifications, only rules on marketing of agricultural products and functioning of the agricultural sector; moreover, the amending regulation calls for modification of the regulation about the use of geographical names in marketing food products other than wine (Regulation (EU) 1151/2012) and two regulations concerning some specific restricted areas in the EU^24^.

In CAP Strategic Plans, MSs have to establish an intervention strategy—made up of the already operating tools of direct payments to farmers, rural development measures and many types of specific intervention in agricultural markets—in which quantitative targets and milestones shall be set to achieve the specific CAP objectives. Such an intervention strategy has to be based on a SWOT analysis carried out on each specific CAP objectives aimed to identify the needs for each of the objectives. Actions considered in the CAP Strategic Plan are financed by the same funds currently operating (EAGF and EAFRD). With regard to the five sectors (fruit and vegetables, apiculture products, wine, olive oil and table olives, hops) for which an aid scheme is currently operated, the proposal confirms a set of sectoral types of intervention associated with sectoral sets of objectives, each of them related to one or more of the specific CAP objectives. MSs, within the CAP Strategic Plan, have to plan, sector by sector, which of the allowed interventions to make available for concerned actors, considering which are the specific CAP objectives and sectoral objectives they have to pursue, and they must do so in relation to the planned management of income support and rural development measures within a perspective of complementarity and subsidiarity. Preliminary decisions concerning the target objectives, being the result of needs identification, are one of the first steps in drawing up the CAP Strategic Plan.

National intervention for fruit and vegetable and apiculture sectors are mandatory for every MS, the wine sector is mandatory for producing countries, the others are optional; in addition, MSs have the opportunity to make interventions available for other products to be defined, obtaining resources from a reduction in the allocations for direct payments (max 3%).

The drawing up of the CAP Strategic Plan is characterised by two important prescriptions. First, it has to comply with the increased ambition with regard to environmental and climate-related objectives that should characterise the CAP beyond 2020, and MSs therefore have to explain in their CAP Strategic Plans, on the basis of available information, how they intend to achieve a greater overall contribution to the environmental target with respect to the period 2014–2020 (Erjavec 2020). Second, MSs have to draw up the CAP Strategic Plans with transparent procedures, involving competent authorities on the environment and climate and organising a partnership with relevant public authorities, economic and social partners, bodies representing civil society and those responsible for promoting social inclusion, fundamental rights, gender equality and non-discrimination.

Beyond the structural features of the reform proposal, some procedural aspects are worth mentioning that, at least in the intentions, should characterise the new CAP in implementing a new delivery model (NDM) “to shift the policy focus from compliance to performance, and rebalance responsibilities between the EU and the MS level with more subsidiarity. The new model aims at better achieving EU objectives based on strategic planning, broad policy interventions and common performance indicators, thus improving policy coherence across the future CAP and with other EU objectives”^25^. According to this model, MSs have to produce an Annual Performance Report on output and expenditure as well as distance to targets set for the whole period that can affect financial transfer to MSs. Such an Annual Report, as well as normative actions delegated to the Commission, should be carried out in line with the Interinstitutional Agreement on Better Law-Making of 13 April 2016.

### Wine policy in the new CAP

Therefore, the reform proposal clearly changes the institutional framework in which the next policy for the wine sector will work. As a consequence, although the proposal maintains the tools for the wine sector, with some modifications shown below, and confirms the asset of a pre-allocated amount of resources, the sectoral intervention—the hard core of wine policy—will no longer be planned in an isolated manner, driven by the current NSP. The new proposed rules (COM/2018/392 final) call for an integration of the sectoral intervention planning with the management of rural development measures and of income support, with the aim of accomplishing the nine new specific CAP objectives and sectoral objectives (Table 8). In addition, the implementing process is characterised by stricter monitoring activity, based on the evaluation of a set of indicators specific for each objective in order to make the results of the policy action evident.

**Table 8:**
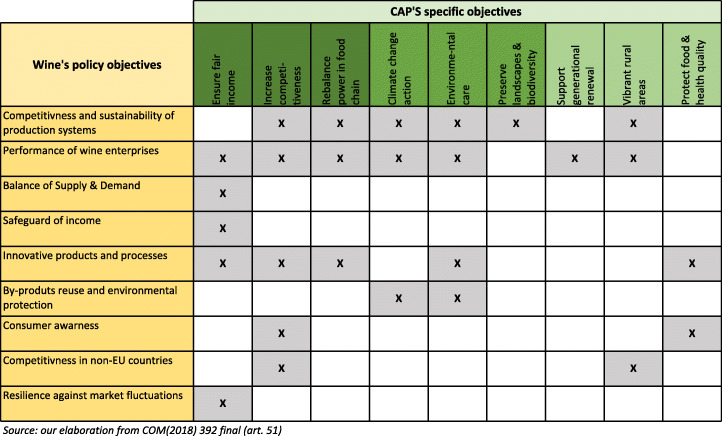
New CAP and wine policy objectives (2021–2027)

It is worth highlighting that the choice of a substantially conservative proposal in terms of spending measures is justified by the statement included in the Commission Explanatory Memorandum (p. 14; see note 20), where it is claimed “while the successive 2008 and 2013 reforms of the wine policy have overall reached their objectives, resulting in an economically vibrant wine sector, new economic, environmental and climatic challenges have appeared. Therefore, the regulation foresees a number of specific amendments to existing rules to cope with these challenges”^26^.

In the new framework, the forthcoming wine budget will be used by each wine producing MS to target one or more of the nine wine sectoral objectives. Table 8 shows these objectives and, on the basis of the statements included in the proposal of Regulation for the Strategic Plan, how each one relates to the nine specific CAP objectives identified. In programming wine sectoral interventions, MSs have to initially select the sectoral objectives to consider, taking into account results of SWOT analysis conducted for drawing up the CAP Strategic Plan. Then, MSs have to decide which type of intervention to make available for the national sector, choosing among a list of nine. Table 9 shows these possible interventions and their relationships with the wine sectoral objectives.

**Table 9:**
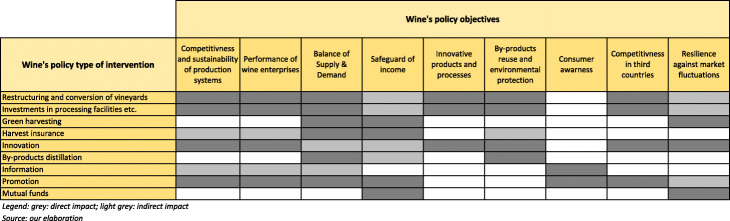
Wine sectoral policy objectives and sectoral types of intervention (2021–2027)

The sectoral types of intervention substantially replicate the actions financed by the measures currently in force (the “The hard core of the wine policy: wine in the single CMO” section, Table 3), with the only innovation represented by the splitting of the current measure about promotion in two types of intervention: (i) the information action within the EU, oriented to the development of awareness of EU consumers about responsible consumption of wine or improvement of knowledge about schemes covering designations of origin and geographical indications; (ii) the promotion of EU wines in non-EU countries.

Concerning the promotion in non-EU countries, the proposed new regulation does not establish any restrictions about wine type but generically refers to EU grapevine products, presuming that all wines, including those without any indication of origin or variety, could be the subject of promotion initiatives in non-EU markets. This is quite an important change, as in the past, the focus of the policy was strictly on PGI/PDO wines. Interestingly, the proposal details, more than for other measures, the actions that could be funded by the measure for promotion; beyond the typical promotional actions and market analysis, an action is included aimed at the preparation of technical files, including laboratory tests and assessments, oenological practices limitations, phytosanitary and hygiene rules, as well as other non-EU country requirements for imports of wine sector products, to facilitate access to non-EU markets.

The intervention supporting the information actions inside EU about PGI/PDO wines and encouraging responsible consumption of wine, which are currently very scarce, in the policy framework proposed by the reform could receive more attention from MSs and wine actors. Indeed, the reform propose as explicit sectoral objective the consumer awareness and, moreover, the information about PGI/PDO wine could help the strengthening of EU wine supply in the domestic market to sustain the internal production from the competitive pressure of other producing countries. However, the success of this intervention could be hampered by restrictive national rules concerning communication about wine.

The reform proposal confirms as possible interventions the support of green harvesting, insurances and mutual funds. As shown in the “The hard core of the wine policy: wine in the single CMO” section, the resources destined to these risk management tools have been always very scarce; the discussion of the reform will be—and likely should be—the occasion to understand if it would be necessary to implement complementary actions to facilitate the adoption of such tools, that could assume more importance in the future.

The reform proposal establishes rules concerning financing of the different types of intervention but does not include rules concerning beneficiaries and their functioning, unlike from the current normative pattern. This is a field, according to the principle of subsidiarity, where MSs should be free to establish rules according to their needs and specific wine sector structure; otherwise, the Commission will detail all operative features concerning the sectoral intervention through a delegate regulation.

Actually, concerning rules on the marketing of agricultural products and functioning of the agricultural sector, the reform, through the proposal of the Amending Regulation (concerning changes to Regulation (EU) 1308/2013), includes relevant novelties related to the fulfilment of new emerging challenges.

From this perspective, a significant proposal concerns the rules about the classification of wine grape varieties with the aim, as declared in preliminary statements of the proposal of the Amending Regulation, of improving the environmental sustainability of EU wine production, allowing grape producers to use varieties with a higher resistance to diseases and that are better adapted to changing climatic conditions.

Currently, all EU wines may be produced with varieties belonging to the species *Vitis vinifera*; moreover, non-PDO wines may also be produced with varieties resulting from a cross between *Vitis vinifera* and other species of the genus *Vitis*, with the exclusion of Noah, Othello, Isabelle, Jacquez, Clinton and Herbemont. For non-PDO wines, the reform proposal also authorises grape varieties belonging to *Vitis labrusca*, or varieties coming from a cross between *Vitis vinifera*, *Vitis labrusca* and other species of the genus *Vitis*, without any restriction. In addition, the reform proposal allows, in the production of PDO wines, also use of varieties coming from a cross between *Vitis vinifera* and other species of the genus *Vitis*.

Actually, these changes in regulation, combined with the confirmed measures concerning the support for restructuring and conversion of vineyards, open a potentially significant evolution of production potential composition, more consistent with sustainability goals.

Relevant changes in sector regulations involve other aspects of the normative side of wine policy.

Concerning PGI/PDO wines, the reform proposal also includes (i) a modification to the definition of designation of origin, in order to achieve better consistency with article 22 of the Agreement on Trade-Related Aspects of Intellectual Property Rights (TRIPS); (ii) some procedural changes in order to allow streamlined approvals of protection requests, with shorter timelines, and rational use of resources, in line with the twin principles of subsidiarity and proportionality; (iii) other changes are proposed in order to obtain a stronger protection of PGI/PDO against counterfeiting on the internet and on goods in transit.

Among rules modifications, it is worth reporting the inclusion—in the range of vitivinicultural products regulated by CAP—of products obtained with a de-alcoholising process, where the alcoholic strength is reduced by more than 20% by volume compared to its initial strength. This inclusion, motivated by the aim to enlarge the options available for a profitable use of grape production, should become operative taking into account the definitions set out in the Resolutions of the International Organisation of Vine and Wine concerning these products.

With reference to new vineyard plantings, the proposal offers to MSs the opportunity to choose between two alternative calculation methods for defining the maximum yearly number of hectares for authorizations: either 1% of the total area actually planted with vines in their territory, as measured on 31 July of the previous year (corresponding to the current system), or 1% of an area comprising the area planted, as measured on 31 July 2015, and the area covered by the old planting rights granted to producers and available for conversion into authorizations on 1 January 2016. This new rule has been proposed with the goal of avoiding the potential loss in production due to the tendency, recorded by European Commission in the years 2014–2017, towards a decrease in the area planted with vines in several MSs.

The proposed adjustment in the regulation of vine planting looks really marginal and unfit to prevent the irreversible decline of the EU area under vine that the current rules may determine^27^. The key issue is that, after the grubbing up of a vineyard, if the area is not replanted in indicated terms, it occurs an irreversible reduction of the production potential with also a consequent decrease in the computation of the annual new available authorisations (1%). This could determine a decrease of new authorisations to a level that do not offset the grubbed up areas. For these reasons, some wine actors are asking for re-establishing a mechanism to create national or regional reserves of authorisation, fuelled with those expired because not used by the entitled farmers. These reserves could satisfy the enlarged demand for authorisations in case of enlarging of market opportunities, with positive effects of the competitive performance of EU wine supply.

The reform proposal ignores the ongoing process which should draw the wine label near to the pre-packed foods, including information about nutritional values and ingredients. Such process started with Regulation (EU) 1169/2011 about food labelling, which called for a re-examination of wine (and alcoholic beverages) labelling rules (Annunziata et al. 2016). In a report published in March 2017, the European Commission concluded that a further extension of current regulation could not be justified and invited alcohol producers to come up with a self-regulatory proposal. The representative bodies of the EU alcoholic beverages sector (wine, spirits, beer etc.) have submitted a proposal to the Commission that is currently under examination (Pabst et al. 2019). The representatives of wine producers have proposed some specific amendments to CAP reform texts to provide wine specific norms, to deal with the difficulties originating by the peculiar wine production process.

### Potential impacts of the proposed reform on the EU wine sector

As illustrated in the previous section, concerning the wine sector, the reform of the CAP currently in progress should result mainly in a reorganisation of the already-operating policy tools and of their management in a unitary frame driven by a strategic approach, with a reduced red tape burden. Using a computer science metaphor, the old hardware represented by the traditional policy measures should be run by a new, more efficient, software.

The CAP Strategic Plan should allow better policy action for the wine sector as a result of policy planning and administration based on an intensive interaction between policy makers and industry actors. Such interaction should be designed to (i) conduct a thorough analysis of the wine market and the evolution of the opportunities for the different segments of wine supply in each MS; (ii) identify the constraints that hamper the full exploitation of human and natural resources and focus the possible role of different types of producers and territories; (iii) finally, share well-defined strategic objectives.

Indeed, such conditions should allow for a manoeuvring of the policy measures based on a sound application of the principle of complementarity making possible a more effective policy action. In particular, the joint planning of sectoral and rural development measures should allow for a rational delimitation of the use of the different tools, avoiding overlapping and the problems mentioned in the “The current support to the wine sector within the CAP” section.

The new CAP was designed taking into account a scenario very different from the one that will arise in the coming years due to the COVID-19 outbreak; in that scenario the proposed objectives would have likely been achieved. Nevertheless, the opportunity offered by the strategic planning required by the reform project should likely facilitate, in a situation that will be characterised by conditions very different in each MSs, the search and implementation of the best strategies to overcome the issues that will affect the agri-food markets in the coming years.

Therefore, in the wine sector as well, the possibility to implement ad hoc strategies, combining the different available tools, should offer more chances—once the most critical phase of the crisis has passed^28^—to deal with the extraordinary challenge to rebuilt the wine market and, in particular, to cope with the two critical areas identified in the performance evaluation of the EU wine sector (see the “The performance of EU wine policy: an analytical evaluation” section): the not entirely satisfactory performance in both contributing to balanced territorial development and reducing the environmental impact of agriculture. Both represent issues even more significant with respect to the objectives defined by the reform for the CAP post-2020.

As a matter of fact, the current objective of balanced territorial development (see Table 2) corresponds in the reform proposal to the objectives “vibrant agriculture” and “generational renewal”. In drawing up the CAP Strategic Plan, it seems possible to better plan well-structured strategies, consistent with the different structural situations existing in declining vitivinicultural areas, as well as to support the segments hit by the COVID-19 crisis, which are those more dependent by the on trade consumptions. In districts where the reduction in the area under vine is determined by very difficult cropping and structural conditions (vineyards being located in marginal areas and/or on steep slopes, small households), the joint planning of sectoral intervention and rural development measures should encourage and consequently enlarge the production of premium wines, which can command prices consistent with high production costs. In particular, the rural development policy could integrate the sectoral measures with support for the development of small farms, involving young farmers and delivering suitable technical and market knowledge. Moreover, the CAP Strategic Plan could make these areas a priority in the distribution of planting authorizations.

In other cases, the reduction in the area under vine happens in areas (mainly in Italy and Spain) with rather good structural conditions, large production capacity and a long tradition of viable production of non-premium wines, which now appear disconnected from the most profitable international value chains. In such situations as well, which are the most relevant in quantitative terms, joint planning of sectoral intervention and rural development measures in drawing up the CAP Strategic Plan could make possible the revitalization of the vitivinicultural activity. While the sectoral intervention could directly support the improvement of cost control in production, logistic facilities and image, the rural development measures could foster the integration among local actors to reach better scale conditions and, possibly, the establishment of partnerships with producers or traders better connected with international distribution networks, located in other regions.

In the implementation of the revitalisation strategies here outlined, a key role may be played by the possibility for involved winegrowers to adjust the size of their vineyards to the needs of new profitable activities. From this perspective, the authorization scheme appears as a factor able to hamper the possibility to revitalise some marginal area levering on vitiviniculture or to revamp the vitivinicultural activity in regions where it is necessary to redefine the relationship with the market. The current scheme, therefore, may weaken the possibility to contribute to the fulfilment of the objectives related to the stability of rural areas that the new planning approach may, on the contrary, sustain.

The issues covered in the current CAP by the objective of sustainable management of natural resources (see Table 2) are, as previously mentioned, of great importance for the reform proposal and covered by the new objectives “climate action”, “environmental care” and “preserve landscape and biodiversity”, objectives which have assumed a higher political relevance after the launch of the European Green Deal. The new policy framework offers the opportunity to coordinate the sectoral intervention, which should be better oriented to a structural improvement favourable to the environmental performance of grape production and wine making, with the application of rural development measures. These, in vitivinicultural regions, should grant solid support to the adoption of sustainable practices to achieve measurable results consistent with “more ambitious” environmental targets that characterise the new CAP.

Surely, in the accomplishment of environmental related objectives, important support could come from a wider use of the new resistant grape varieties coming from a cross between *Vitis vinifera* and other species of the genus *Vitis* resistant to downy mildew and oidium, which have excellent sensorial properties and, as previously mentioned, are permitted in PDO production by the reform proposal.

But to what extent the wider use of resistant varieties will occur, and at which rate, is currently unpredictable. Beyond what EU allows in general, the Member States, entitled to classify which wine grape varieties may be planted on their territories for the purpose of wine production, have very different positions with respect to new hybrids; the same is true within some wine producing countries. In particular, the use of these new varieties in PDO wine production raises many doubts, at least in Mediterranean countries, as the use of only grapes from *Vitis vinifera* is one of the main elements which historically has defined the concept of designation of origin (Meloni and Swinnen 2016; Meloni and Swinnen 2018). Moreover, also when one or more of these new varieties are allowed in PDO and PGI wine production by competent national or regional authorities, their actual use requires a change in the product specification^29^, which can happen only after a thorough evaluation of the oenological potential in the specific cropping conditions of the geographical area concerned^30^.

Such new varieties are increasingly available and actively promoted by an international association, the PIWI association. Therefore, beyond their classification as common wines, varietals, PGI or PDO, wine based on the new hybrids, under the pressure of consumer preference for wines produced without pesticides and social pressure concerning environmental protection, could become a specific new segment of European wine supply, deeply modifying the current competition scenario (Montaigne et al. 2016). Most likely, the discussion of the reform proposal will provide an opportunity to arbitrate between the different points of view on the use of new resistant varieties; but probably such discussion will also include the topic of new varieties obtained with new breeding techniques as genome editing. Such new breeding techniques could in the near future be accepted by the European Commission in the framework of the implementation of the “Farm to Fork” strategy (Bird 2020).

## Conclusions

Up to the 1980s, the wine producing countries in EU were the only key players in the international wine market. Later, as the globalisation process took place, they successfully sustained the catch-up process of the wine producing countries in the New World, but now they have to deal with a market where the competitive pressure of the new comers is very high. The reasons behind the success of EU wine supply are certainly many and it is questionable which has been, and currently is, the effect of the EU wine policy on the performance of EU wine producers. However, as the wine policy is going to be reformed as part of the more general agricultural policy of the EU (CAP), this paper provides a comprehensive presentation of how CAP evolved in terms of objectives and tools over its history and, with specific reference to wine, which is the design, the rationale and the potential effect of the reform in light of some critical issues related to the current performance of the EU wine sector.

The CAP has been considered for long time as the cornerstone of the European integration process. The gradual broadening of its original objectives has led to a progressive reshaping of the policy tools, which have resulted in a very large legislative framework, implemented through three main lines: farmer income support; expenditure and regulatory measures to stabilise or develop agricultural markets (the so-called single CMO); measures for rural development.

Wine policy, after a radical process of reform started in 1999, deepened in 2008 and lead up in 2013, currently stems from the three main CAP lines and is characterised by a particularly significant number of regulatory measures—encompassing grape cultivation and processing, wine presentation and commercialisation—and by expenditure measures destined to the whole supply chain. As a matter of fact, wine policy is the only true vertical policy within CAP. This aspect takes its roots in the peculiar nature of the wine production process, where the value of a perishable agricultural raw material—the grape—is strictly dependent on processing opportunities.

The current wine policy objectives appear fully coherent with those identified in 2013 for the general CAP: viability of food production; sustainable management of natural resource; balanced territorial development. However, expenditures and regulatory measures operating for the wine sector do not have allowed a balanced pursuit of all these goals. Analysing the general performance of the wine sector in the new century, it comes to light that only the objective of the viability of production appears almost accomplished. However, risks are emerging for the future, coming from the economic crisis caused by the COVID-19 outbreak, which worse the issue of the exit of the UK from the EU.

The current CAP objectives have been taken over by the reform proposal for the post-2020 period, with an explicit commitment for ambitious environmental results. New opportunities for a more balanced achievement of all policy objectives should derive from the implementation of the most significant novelties proposed. The proposal includes a more articulated set of general and sectoral objectives, largely consistent with those currently in force, and confirms the toolbox available for the wine sector, with some interesting modifications. It is worth to be highlighted the proposed wide reorganisation of the joint management of CAP tools (included those for wine), whose implementation should be driven by a strategic approach, respecting the principle of complementarity. In a nutshell, traditional and new measures should be run in a more efficient and monitored programming framework: the so called CAP Strategic Plan.

Observing the whole of changes which should be delivered by the next reform, it seems possible to highlight two main opportunities and two evident limitations.

The first opportunity is related to the environmental issues, whose relevance has been strengthened even more by European strategy of sustainable growth announced with the European Green Deal. The declared objective of the reform project is to pursue a higher level of climate and environmental ambition. In this context, wine actors should find larger opportunities to reduce their environmental impact, preserving at the same time their compliance with the social and economic aspects of sustainability. In such a way, responding to the increasing demand for sustainable wines, they could improve their competitiveness.

The second opportunity is related to the role that the vitivinicultural activity could play in maintaining or restoring the socioeconomic equilibrium in some problematic areas. As a matter of fact, this activity was until now one of the more profitable in agriculture and in some cases the only one able to generate reasonable revenues. The combined use of the different CAP tools—direct payments, rural development measures, sectoral measures—should allow in such problematic areas the development of new supply chains effectively connected with profitable markets.

On the side of limitations, the first one is related to the authorisation scheme. If, on one hand, the control of the area under vine is useful to avoid the return of structural surpluses, the proposed marginal changes to the current rules are clearly unfitted to avoid the depletion of the production potential and the rigidity of the authorisation scheme hinder the possibility to revamp vitivinicultural activity in problematic areas, in which this could be possible with appropriate programmes.

The second one is related to the lack of proposals concerning the wine labelling of ingredients and nutritional facts. However, the lift of a secular privilege of wine labels will be likely included in the further discussion of the reform project in the coming months.

In the hypothesis that the COVID-19 outbreak will not cause a radical change in the global agri-food system, and an extraordinary immediate support by EU and MSs will preserve the present structure of the EU wine industry, the proposed reform could potentially support the EU wine sector in maintaining profitability and market shares. More generally, it could contribute to the economic and social wellbeing of the rural areas involved. The reform project outlines a complex planning process, which calls for a strong commitment of public institutions and private stakeholders; furthermore, the intended improvement of the administrative processes (the new delivery model) of the policy intervention looks very ambitious. The risk is high that the exercise of drawing up the CAP Strategic Plan will produce a document only formally compliant with the prescriptions and that the deployment of the new delivery model of agricultural policy will result in a *maquillage* of the current routines. Only when the renewed CAP is fully activated it will be possible to understand if the reform project will be effective in the achievement of stated objectives and whether it will be efficient and able to eliminate the weaknesses highlighted by some authors and mentioned in the introduction.

This paper intended to offer a comprehensive overview of the current status of EU wine policy, as part of the CAP, and a perspective on the evolution of such a policy. The paper fills a gap in the academic literature but suggests further interesting research avenues. These may include a detailed quantitative analysis of the cause-effect relationships between single support interventions and performance, at least in specific test areas, and an analysis of the effect on EU wine performance of the sectoral regulation, which includes a wide array of rules on production and geographical indication. Finally, considering the many criticisms addressed to the EU wine policy and, in particular, the current accelerations in the evolution of the international scenario, an exploration of the possible effects of radical changes in the policy approach would be welcome, leading to a truly alternative regulatory framework.

## Data Availability

The datasets used and/or analysed during the current study are available from the corresponding author on reasonable request.

## Abbreviations

CAGR: Compound annual growth rate
CAP: Common Agricultural Policy
CMO: Common Market Organisation
EBITDA: Earnings before interest, tax, depreciation and amortisation
EAFRD: European Agricultural Fund for Rural Development
EAGF: European Agricultural Guarantee Fund
EU: European Union
FADN: Farm Accountancy Data Network
IBO: Interbranch organisation
MFF: Multiannual Financial Framework
MS: Member State
NSP: National Support Programme
OECD: Organization for Economic Co-operation and Development
PDO: Protected Designation of Origin
PGI: Protected Geographical Indication
PO: Producer organisation
TRIPS: Agreement on Trade-Related Aspects of Intellectual Property Rights

## Footnotes

1 The principle of subsidiarity is defined in Article 5 of the European Union Treaty. It aims to ensure that decisions are taken as closely as possible to the citizen and that constant checks are made to verify that action at the EU level is justified in the light of the possibilities available at national, regional or local levels.

2 Founders: Italy, Spain, France, Luxemburg, Tunisia, Hungary, Greece and Portugal; currently 46 member countries.

3 Founders: Argentina, Australia, Canada, Chile, New Zealand, South Africa, the USA.

4 In the considered period, European agriculture has experienced deep social and economic transformations, that can be summarised as follows: the serious reduction of the role played in the economy, in relation to the share on GDP, employment and trade; the application of relevant changes in the methods of production, with the spread of machines and new technologies; the increase in yields and the progressive specialisation of production; the impressive exodus from the rural areas and the related ageing of farmers (Fennel 1987).

5 A “regulation” is a binding legislative act which must be applied in its entirety by the EU MSs. The “directive” is a legal act which sets out a goal that all EU MSs must achieve; however, it is up to each country to devise its own laws to reach this goal.

6 In the second part of the 1960s and at the beginning of 1970s become evident that the wine production capacity in the largest European traditional producers, France and Italy, was increasingly exceeding the domestic consumption and that, therefore, the export of wine emerged as a strategic need to maintain the sustainability of vitivinicultural activity; from the qualitative point of view, the domestic demand and the rising external demand was characterised by new and increasing quality requirements (Anderson and Pinilla 2018).

7 In the past, each market sector was regulated by a sectoral Common Market Organisation (CMO) defined by specific expenditures, measures and marketing rules established by separate regulations. The Regulation (UE) 1308/2013, currently in force, is the result of a sequence of reforms and of the transformation of twenty-one sectoral regulations in a single one. For this reason, the normative framework established by this regulation is appointed as a “Single Common Market Organisation” (Single CMO).

8 The mechanism of support based on the NSP was already established by the 2008 reform.

9 See notes 11 and 12.

10 Regulation (UE) 1308/2013 includes basic provisions concerning oenological practices; these are integrated by the delegated Regulation (EU) 934/2019 (which update the Regulation (EC) 606/2009) and, as concerns organic wine, by the Regulation (EU) 203/2012.

11 According to Regulation (EU) 1308/2013, and following a long tradition, a geographical name used to identify a wine is recognised and protected by EU on request of the concerned producers. The use of the name requires to produce the wine respecting a self-established product specification which defines the production-area boundaries, admitted grape varieties, maximum yield, analytical parameters of wine and other aspects of grape production, processing and wine finishing; therefore, part of the rules that EU wine producers have to respect are voluntarily defined and not imposed by EU rules. Rules concerning geographical names for wine differ from those laid down for other agriculture products and foodstuffs in Regulation (EU) 1151/2012 but consistent in terms of general principles.

12 According the Regulation (EU) 1308/2013, art. 93: “a designation of origin” means the name of a region, a specific place or, in exceptional and duly justifiable cases, a country used to describe a wine which fulfils, among others, the requirement that the quality and characteristics of the concerned wine are essentially or exclusively due to a particular geographical environment with its inherent natural and human factors; “a geographical indication” means an indication referring to a region, a specific place or, in exceptional and duly justifiable cases, a country, used to describe a wine which fulfils, among others, the requirements that it possesses a specific quality, reputation or other characteristics attributable to that geographical origin.

13 Regulation (UE) 1308/2013, art. 167.

14 IBOs related to specific PGI/PDO are: *Comité Interprofessionnel* and *Syndicat d’appellation* in France; *Consorzio di Tutela* in Italy; *Consejo Regulador* in Spain. Moreover, in Spain operates also a national IBO related to the whole production: *La Interprofesional del Vino de España* (OIVE).

15 As results of the compromise between the opposite positions, it is worth to underline that the new scheme—unlike the previous regime of “planting rights” in force between 1983 and 2015—is based on a mechanism that does not allow the transfer of authorisations among wine growers, by payment or for free, so as to avoid speculation.

16 With the 2008 reform (Regulation (EC) 479/2008), some of the resources of the wine budget for market measures in the CMO were transferred in favour of the rural development fund, aiming to support the wine sector in different areas of intervention (young farmers, investments in technical facilities, marketing improvements, vocational training, support for producers' organisations after entering quality schemes, agro-environment support, early retirement). It is therefore a paradox that a share of RDP resources were not reserved systematically for wine producers in the concerned MSs or regions.

17 According to these data, the support to EU wine producers looks very generous, and these authors claim “it is little wonder that other wine-producing countries worry about their ability to compete in international markets against supported EU producers” (p. 297).

18 Despite the reform of 2008 and the modifications that occurred in 2013, the results of Anderson and Jensen, based on average data that include also a short period preceding the introduction of NPS, in aggregate terms can be considered largely still valid. However, it must be underlined that their estimations are based on the strong assumption that the wine sector has benefitted from a share of rural development resources equal to its weight in overall agriculture. In addition, also included in the computation national resources independent of CAP (such as research and training), which are outside the purpose of this work.

19 A similar distribution of resources occurred in the previous implementation period of NSPs (2009–2013).

20 It is worth to underline that, starting from the entry in force of the new scheme of authorizations, data shown only a limited recovery of total European area under vine. In particular, the annual increase recorded is considerably lower of those expected on the base of the information about the implementation of the new rules in force (European Commission 2019). These differences could be related to two different factors: (i) a misalignment in the time of using the new plantings authorised (authorizations have a duration of three years, before expiring), with the result of a delayed visibility of the impact produced; (ii) the contextual loss of vineyards within specific producing regions (put in evidence by the analysis of national inventories), characterised by a persistent phenomenon of abandonment, that is hardly compensated by the new authorizations, mainly utilised in the more dynamic areas.

21 FADN is an EU instrument for evaluating the income of agricultural holdings and impacts of the CAP. The FADN was launched in 1965, with Regulation 79/65/EEC that established the legal basis for the organisation of the network.

22 Since 2012, with Regulation (EU) 203/2012, which defines and regulates organic wine production, it is possible to sell “organic wine” and no longer wine produced from organic grapes.

23 The amending regulation is also defined as a regulation of the Common Market Organization modifying the regulation that currently defines the single CMO. The two mentioned proposals are accompanied by a third instrumental proposal for a horizontal regulation (COM/2018/393 final) that defines the general framework of CAP financing, management and monitoring (repealing the current Regulation (EU) 1306/2013).

24 Regulation (EU) 228/2013 and Regulation (EU) 229/2013.

25 Explanatory memorandum to reform proposals (p. 2) (European Commission 2018b).

26 Such arguments are also consistent with the last evaluation of CAP measures applicable to the wine sector (Agrosynergie 2018).

27 In the same direction, an outlook of European Commission foresees a progressive reduction of area under vine (European Commission 2017c).

28 For this to happen without profound changes in the current structure of EU wine industry, extraordinary financial support from the EU and MSs will be needed.

29 See note 11.

30 As a matter of fact, the administrative pathway for the admission of these new resistant varieties into wine production could be facilitated by their direct classification as *Vitis vinifera*, as their genotypes derive more than 95% from *Vitis vinifera*; this is already happening in Germany and Central Europe but not in France and Italy. But this would be likely insufficient for an easy adoption by winegrowers. These new varieties are the result of a deep change in the technological paradigm of plant breeding, and their acceptance by producers looks inevitably like a rather slow process (Montaigne et al. 2016; Barker 2017).
